# Are Baby Rattlesnakes More Dangerous than Adults? Origin, Transmission, and Prevalence of a Media-Driven Myth, with Evidence of Effective Messaging to Dispel It

**DOI:** 10.3390/toxins18030144

**Published:** 2026-03-14

**Authors:** William K. Hayes, M. Cale Morris

**Affiliations:** Department of Earth and Biological Sciences, Loma Linda University, Loma Linda, CA 92350, USA; mmorris@students.llu.edu

**Keywords:** Viperidae, ontogeny, envenomation, venom-metering, venom yield, journalism, environmental education, effective messaging

## Abstract

The easily defanged myth that baby rattlesnakes (genera *Crotalus* and *Sistrurus*) are more dangerous than adults has persisted in North America despite all evidence to the contrary. The most often cited reason for the babies-more-dangerous (BMD) myth is the venom-dump (VD) hypothesis: babies, in contrast to adults, cannot control how much venom they expend, and therefore inject all of it when biting. We undertook three approaches to explore the origin, transmission, and prevalence of this myth and its most frequent explanation. First, we examined historical newspaper accounts. From 130 newspaper stories mentioning the relative danger of baby rattlesnakes, we identified a timeline in which (1) most stories prior to 1969 were factually correct; (2) the BMD myth and VD hypothesis likely originated in the mid-to-late 1960s and became entrenched in California, especially, from 1970 to 1999; (3) factually incorrect statements subsequently prevailed throughout North America from 2000 to 2014; and (4) factually correct stories regained prominence with apparent effective messaging success from 2015 onward. We further learned that general information stories about rattlesnakes, more often citing subject experts like university professors, were much more likely to provide accurate information than local snakebite stories, which more often cited health professionals (e.g., physicians, veterinarians, pharmacists) and emergency responders (e.g., police and fire officers) who frequently supplied misinformation. Second, we surveyed familiarity with the BMD myth and VD hypothesis among 53 university classrooms (including one high school) representing 3751 students across 29 states within the United States. Consistent with the California media’s outsized influence on misinformation transmission, familiarity with the myth was greatest in the southwestern states (52.6%) and declined moving north and east, with the least familiarity in the northeastern states (16.4%). Third, a small survey of 75 emergency responders and health professionals from Southern California revealed that a whopping 73.3% actually believed the BMD myth. Numerous organizations generally regarded as authoritative further amplified the misinformation, especially on the internet, where some content persists to this day. Unfortunately, belief in the BMD myth and VD hypothesis can lead to negative consequences, including misinformed risk-taking by those encountering snakes, unwarranted fear among snakebite victims, and inappropriate care delivered by misinformed or patient/family-pressured medical professionals. Our findings target health professionals and emergency responders as priority audiences for education.

## 1. Introduction

Myths regarding rattlesnakes (genera *Crotalus* and *Sistrurus*) are widespread and have a long-standing presence in North American culture [1,2,3,4]. They persist across generations via conversations, news outlets, and online platforms. Advancements in communication technology and social media enable myths to form and spread more quickly [5,6,7,8,9]. Rattlesnake myths often center on misunderstandings of rattlesnake behavior. Examples include being especially aggressive, chasing people, and always giving an audible rattle warning when nearby or before they strike [1,2,3,4,10]. Misconceptions about rattlesnakes create unnecessary fear and frequently result in people harming or killing them [11]. Rattlesnakes occupy an important role in the ecosystems they dwell in, and in recent years, their populations have dropped significantly in many parts of the United States [12,13].

Rattlesnakes possess a sophisticated system for delivering venom, which helps them efficiently kill prey [14,15,16,17,18,19]. This mechanism also serves as a protective strategy, allowing them to defend themselves against threats such as predators and humans. In North America, a widespread myth persists that baby (or neonate or juvenile) rattlesnakes are more dangerous than adults [20]. The perception stems largely from the belief that babies cannot control how much venom they expel when biting, and therefore deliver all of it. Conversely, adult rattlesnakes are often seen as less threatening despite their size because they are more adept at controlling their venom expenditure. However, substantial evidence from three lines of inquiry refutes this myth, which in our experience has become one of the most frequently repeated falsehoods about snakes in the United States.

First, baby rattlesnakes possess far less venom than adults. Rattlesnakes synthesize and store venom within a pair of venom glands in their head. As the snakes grow in length, a single dimension, their venom gland expands in three dimensions. Thus, the volume of venom in the gland increases exponentially as the snake grows in length [21,22,23]. Adult rattlesnakes, accordingly, possess significantly greater quantities of venom compared to juvenile snakes.

Second, experimental studies of venom expenditure confirm that adult rattlesnakes do indeed expel substantially more venom than babies when biting [14,20,24]. Two studies have demonstrated this for predatory bites [22,25] and another for defensive bites [20]. Moreover, another study suggests that juvenile rattlesnakes, like adults [8,9,26,27], possess cognitive control of venom expulsion, as they deliver more venom into larger prey than smaller prey [28]. This capacity for venom metering presumably applies to defensive bites as well. Adults do not expend all their venom in a single bite and can withhold some for subsequent bites [14,24,25]. Our own observations during venom extractions suggest the same is true for baby snakes.

Third, clinical studies have shown that baby rattlesnakes cause less severe envenomations of humans than adult snakes [29,30,31]. These less severe envenomations occur even though the juveniles of many (but not all) species possess more toxic venom [23,32,33,34,35,36,37,38].

So where did the babies-more-dangerous (BMD) myth come from, and how did it become so prominent? To explore the origin, transmission, and prevalence of this myth, we examined historical newspaper accounts across North America and surveyed student familiarity with the myth across the United States. We also conducted a small survey of Southern California emergency responders and health professionals. Our findings draw attention to a remarkably common media-driven myth, routes of transmission, and a disturbingly high prevalence of belief. The findings also suggest that misinformation can eventually be dispelled with some success, and they identify prominent sources of misinformation that should be targeted by educational campaigns.

## 2. Results

### 2.1. Assessment of Newspaper Stories: Historical Origin and Transmission of the BMD Myth

Our detailed searches of current and historical newspaper archives identified 130 stories (126 from the United States, four from Canada) that addressed the BMD myth. Although we found stories as early as 1907 comparing the relative danger of baby and adult rattlesnakes, the first mentions we found of baby rattlesnakes being more dangerous were in 1936, 1958, and 1965 (Supplementary Materials Table S1). From 1967 onward, numerous stories claimed that baby rattlesnakes were more dangerous (Supplementary Materials Table S1). Subjective assessment prompted us to consider three factors regarding factual correctness of the stories, (1) temporal trends, (2) regional variation among the media sources, and (3) context of the story, which influenced the choice of authorities cited. We therefore conducted a binary logistic regression analysis to assess how year (1900–2025), location (California versus other regions of North America), and context (general information or recent envenomation) influenced whether the story was factually correct (babies are not more dangerous) or incorrect.

**Figure 1:**
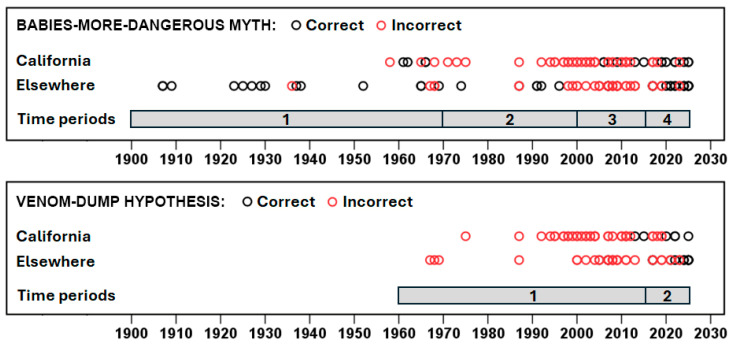
Timelines and factual correctness of newspaper stories mentioning the babies-more-dangerous (BMD) myth (bites by baby rattlesnakes are more deadly) and the venom-dump (VD) hypothesis (babies, unlike adults, dump all their venom in a single bite) for two regions of North America (California and elsewhere). Both the myth and the hypothesis are factually incorrect: baby rattlesnakes are less dangerous because they possess less venom in their smaller glands and inject less when biting, and baby rattlesnakes, like adults, are capable of controlling venom expenditure and do not dump all their venom in a single bite. Statistical analyses confirmed that factual correctness changed over time for both the BMD myth and the VD hypothesis, with the pattern corresponding to the time periods indicated. With multiple stories in some years, the black circles for some correct stories are hidden behind the red circles of incorrect stories, including a handful of correct BMD stories from both regions during the period 2004–2015 and three correct VD stories from elsewhere in 2005–2008.

**Figure 2:**
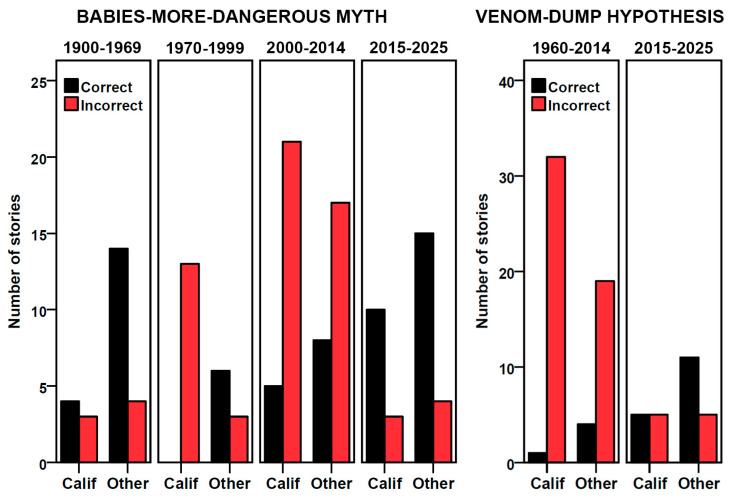
Number of factually correct and incorrect newspaper stories mentioning the babies-more-dangerous myth (**left**) and the venom-dump hypothesis (**right**) that originated in California (Calif) or elsewhere (Other) across relevant time periods. Factual correctness changed over time.

The preferred logistic regression model (likelihood ratio χ^2^ = 38.38, df = 4, *p* < 0.001), as assessed by the Akaike information criterion (AIC), included the main effects and a significant interaction between year and region (Wald χ^2^ = 4.59, df = 1, *p* = 0.032). A competing logistic regression model (ΔAIC = 0.25) included an additional, non-significant context × year interaction but did not improve the model fit or substantially alter the conclusions. Factual correctness among the two regions considered appeared to vary across four time periods (Figure 1 and Figure 2). We established boundaries for the time periods based on the trends we identified in the data and rounding to the decade or half-decade. During the first period, 1900–1969, most stories, regardless of region, correctly stated that baby rattlesnakes were less dangerous (California: 57.1% of 7; elsewhere: 77.8% of 18). During the second period, 1970–1999, no stories from California were correct (0.0% of 13) whereas those from elsewhere were largely correct (66.7% of 9). During the third period, 2000–2014, few stories were correct in either California (19.2% of 26) or elsewhere (32.0% of 25). During the most recent period, 2015–2025, the large majority of stories were correct in both California (76.9% of 13) and elsewhere (78.9% of 19).

**Figure 3:**
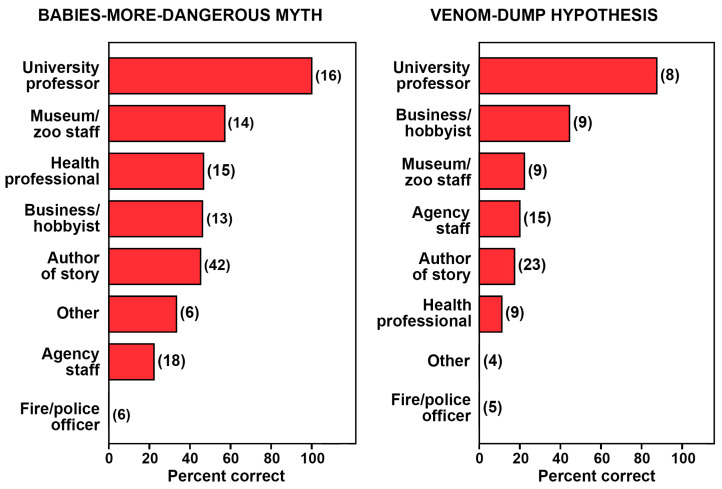
Percentage of correct stories that mentioned the babies-more-dangerous myth (**left**) and the venom-dump hypothesis (**right**) based on the employment category of the authorities cited. The number of news stories for each category is indicated within parentheses. See Methods for examples of employees within these categories.

The preferred model also found the main effect of context to be significant (Wald χ^2^ = 18.97, df = 1, *p* < 0.001). Stories that provided general information about rattlesnakes were more likely to be correct (62.8% of 86) than those that centered on recent cases of snakebite in the community (18.2% of 44). The difference can be attributed in part to the authorities relied on by the authors of the stories: general information stories more often cited university professors (i.e., subject experts; 15.1%) compared to snakebite stories (6.8%), whereas snakebite stories more often cited healthcare professionals (20.5%) and fire/police officials (11.4%) compared to general information stories (7.0% and 1.2%, respectively). A separate chi-square analysis confirmed that the sources cited varied substantially in correctness (χ^2^ = 28.82, df = 7, *p* < 0.001, Cramer’s *V* = 0.47; see Figure 3), with university professors providing the most reliable information (100% of 16 correct), healthcare professionals being less reliable (46.7% of 15 correct), and fire/police officers being the least reliable (0.0% of six correct).

Given the importance of context and cited authority, we suspected that a shift in story-telling and reliance on experts improved the factual accuracy of stories during the most recent time period (2015–2025). Indeed, after the BMD myth became prominent in the 1970s, the proportion of general information stories increased markedly (χ^2^ = 11.59, df = 2, *p* = 0.003, Cramer’s *V* = 0.33), comprising 44.5% of 22 stories, 51.1% of 51 stories, and 84.4% of 32 stories across time periods 2 through 4, respectively (it was also high during time period 1, at 92.0% of 25 stories). Author reliance on authorities also changed, especially during the fourth time period. The proportion of stories citing a university professor increasing significantly (13.6%, 5.9%, and 31.3% of stories; χ^2^ = 9.85, df = 2, *p* = 0.007, Cramer’s *V* = 0.31) and the proportion of stories citing a healthcare professional decreased somewhat, though not significantly, across the same time periods (13.6%, 15.7%, 3.1%; χ^2^ = 3.20, df = 2, *p* = 0.20, Cramer’s *V* = 0.18), respectively.

### 2.2. Assessment of Newspaper Stories: Explanations Used to Support the BMD Myth

The newspaper stories offered three major explanations for whether baby or adult rattlesnakes are more dangerous (see Supplementary Materials Table S1). We phrase these as hypotheses. Table 1 provides some of the more entertaining quotes from the stories.

The aforementioned *venom-dump* (VD) *hypothesis*, mentioned in 82 stories (63.1% of all stories), wrongly asserts that babies cannot control the volume of venom they release when biting and therefore expulse all of it, which makes them more dangerous. This explanation was used wrongly 52.3% of the time to claim that baby snakes are more dangerous. The earliest mention we found of this explanation being used to claim that babies were more dangerous was from a Tennessee newspaper in 1967, which cited a salesman for New York Life Insurance Company who was an active rattlesnake hobbyist [39]. The myth was repeated twice more in 1968 (Alabama) and 1969 (South Dakota) before becoming prominent in California from 1975 onward (Supplementary Materials Table S1).

**Table 1:** Examples of misinformed claims from newspaper stories that baby rattlesnakes are more dangerous than adults (babies-more-dangerous myth) and/or cannot control their venom expenditure when biting (venom-dump hypothesis to explain the myth).

Quote and Authority Cited	Source
“Adult rattle snakes have a little bit more control, so a baby rattle snake may eject a person or an animal that they come into contact with a lot of venom where an adult rattle snake may not do so.” Authority cited: Administrator for the City of Wichita Falls Animal Services.	[40] 2017
“The younger snakes are actually more dangerous than the adults because the adults have more control over how they disperse their venom.” Authority cited: Prosecutor speaking in a court case.	[41] 2013
“Baby rattlers may be even more dangerous than adults…An adult will try to save its venom to immobilize its prey, and may not inject any when defensively striking a human. A juvenile, though, doesn’t know yet how to moderate its venom ‘dose,’ so it could give you the full wallop.” Authority cited: Supervising ranger at Wilderness Park.	[42] 2011
“Young rattlesnakes can be more dangerous as they have not developed the ability to control the flow of venom, meaning all the venom is released during a bite…While an adult rattlesnake can control the flow of the venom it releases, a juvenile will release all the venom it has, which ultimately requires a larger dose of antivenin to treat.” Authority cited: Chief medical officer and ER physician.	[43] 2011
“…baby rattlesnakes are potentially more dangerous than larger ones…adult snakes only release venom 75 percent of the time. ‘Babies its one-hundred percent [envenomation]. You get bit by a baby, it doesn’t save its venom for its prey. It doesn’t know any better. It will invenomate [sic] you every time it strikes, and they can do that several times,’ he said.” Authority cited: Founder of reptile rescue company.	[44] 2009
“While it is true that the venom of baby snakes is more concentrated and therefore potentially more lethal, the volume of venom in these babies is much smaller than in the adults…Even though the baby rattlers release their entire amount of venom with each bite, the total dose received is often less than their adult counterparts.” Authority cited: None.	[45] 2004
“The baby snakes are also more dangerous than the adults. The adult snakes inject only enough venom into their prey as necessary and often do not inject venom into a person. A baby snake doesn’t make that judgment and injects all of its venom.” Authority cited: State Park ranger.	[46] 1995
“…young rattlesnakes are more dangerous, but not because they are quicker. Older snakes develop sphincter control and save some venom for the next bite; young ones give you the whole shot right off—more venom.” Authority cited: None.	[47] 1987
“They are dangerous at birth...more dangerous than the adult snake...very aggressive little fellows who will throw themselves into a defensive pose and strike repeatedly when disturbed. Where the more reserved adult will strike only once or twice, the baby has not yet learned control and will keep on striking, injecting more venom into his unhappy victim...” Authority cited: None.	[48] 1975
“…baby rattlers…are even more dangerous than adult rattlers because when they come out of hibernation their venom is highly concentrated.” Authority cited: Part-time naturalist in Arcadia’s Wilderness Park.	[49] 1967
“Some experts have pointed out…that while a small, young rattlesnake will inject all his venom into whatever it bites, the older, larger ones will inject only about ¼ on the first strike, holding plenty in reserve in case other strikes are indicated. This means…that a bite from a smaller snake might well be more dangerous than one from a larger reptile.” Authority cited: Salesman for New York Life Insurance Co. and a rattlesnake hobbyist.	[40], 1967
“Young rattlers are more dangerous than mature snakes…because they are poisonous from the time they are born and will strike at almost anything.” Authority cited: Police chief.	[50] 1965
“Contrary to popular belief…the bite of a baby rattler is more dangerous than that of an adult snake. The venom is thinner and far more important.” Authority cited: Owner of the Reptile Leather company.	[51] 1936
“Of course, the longer the fangs, the more dangerous is the bite, because the venom is with greater certainty thrown into the arterial bloodstream.” Authority cited: None.	[52] 1929

The *venom-quantity* (VQ) *hypothesis*, cited in 39 stories (30.0% of all), correctly posits that larger snakes possess much more venom than babies and deliver more venom when biting, which makes them more dangerous. This explanation, when expressed, was stated correctly in all but one case (97.4%).

The *venom-composition* (VC) *hypothesis*, mentioned in 14 stories (10.8%), suggests that the higher protein concentration or higher toxicity of baby rattlesnakes makes them more dangerous. Factuality is nuanced for this explanation, as ontogenetic trends in protein concentration remain largely unstudied and are likely trivial [53], see also [54]. Ontogenetic trends in toxicity also vary among species, being highest in the young of many species but sometimes remaining similar to adults [23,32,33,34,35,36,37,38]. Accordingly, we did not assess the frequency of this explanation being correct.

Several additional explanations were offered (Supplementary Materials Table S1), including an enhanced inclination of babies to strike and do so repeatedly (seven mentions), the greater fang length of adults (six mentions), and the absence of a rattle and noise for babies (one story). Although these additional explanations may or may not be true, they have negligible bearing on the relative danger of baby rattlesnakes compared to adults.

We subjected the factual correctness of all mentions of the VD hypothesis to the same binary logistic regression analysis as the BMD myth. The best model included only main effects (likelihood ratio χ^2^ = 36.01, df = 3, *p* < 0.001), with year being the only significant predictor (Wald χ^2^ = 13.82, df = 1, *p* < 0.001). A competing model (ΔAIC = 0.70) included a region × year interaction, but year remained the only significant predictor, as was true for all other more complex models. Correctness differed between two fairly well-defined periods (Figure 1 and Figure 2). From 1967 to 2014, the large majority of mentions (91.1% of 56), regardless of region, falsely claimed that babies were more dangerous. From 2015 through 2025, the majority of stories (61.5% of 26) correctly reported that the hypothesis was wrong and that adults are more dangerous.

Similar to the BMD myth, a chi-square analysis confirmed that the sources cited (collapsed to four categories due to sparse cells) varied substantially in correctness (χ^2^ = 17.87, df = 3, *p* < 0.001, Cramer’s *V* = 0.47; see Figure 3). University professors once again provided the most reliable information (87.5% of eight correct), whereas all other categories of experts were correct <50% of the time. Healthcare professionals (11.1% of nine correct) and fire/police officers (0.0% of five correct) were notable for sharing misinformation. The one story applying the myth incorrectly by citing a university professor [55] actually misquoted one of us (WKH).

Once again, the importance of context (for BMD stories) and cited authority prompted us to consider whether the context of story-telling and reliance on experts improved the factual accuracy of stories during the most recent time period (2015–2025). Indeed, the proportion of general information stories increased from 60.2% of 98 stories before 2015 to 84.4% of 32 stories after (χ^2^ = 6.29, df = 1, *p* = 0.012, Cramer’s *V* = 0.22). The proportion of stories citing a university professor also increased significantly from 8.2% before 2015 to 31.3% after (χ^2^ = 9.14, df = 1, *p* = 0.003, φ = 0.30), and the proportion citing a healthcare professional decreased from 15.1% to 3.1%, approaching significance (χ^2^ = 3.14, df = 1, *p* = 0.077, φ = 0.17).

### 2.3. Survey of University Students: Regional Familiarity with and Prevalence of the BMD Myth

We began assessing newspaper stories in 2007, when online sources and archives had a limited number of stories available. After analyzing the initial sample of 49 stories, we quickly learned that the stories were universally wrong in California and largely correct elsewhere during the period 1970–1999, which led us to suspect that the news media in California played an outsized role in spreading and perpetuating the BMD myth and the VD hypothesis. To evaluate this possibility, we surveyed students in General Biology classes across the United States to assess familiarity with the myth. Cooperating instructors verbally asked students to raise their hands if they had heard that “baby rattlesnakes are more dangerous than adults because they have not learned to control the amount of venom they inject when biting, and therefore inject more.”

**Figure 4:**
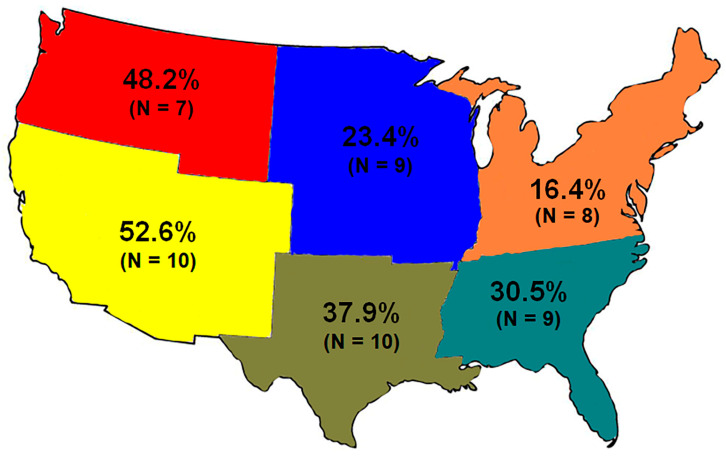
Proportion of university students familiar with the babies-more-dangerous myth across six geographic regions of the United States in 2008–2009. The number of classrooms surveyed in each region is indicated parenthetically. Familiarity with the myth was greatest in the southwest and lowest in the northeast.

We obtained responses from 53 classrooms (52 colleges/universities and one high school), representing 3751 students across 29 states. A one-way analysis of variance (ANOVA) confirmed that familiarity with the myth varied across the six geographic regions (*F*_5,47_ = 8.45, *p* < 0.01, η^2^ = 0.47), as portrayed in Figure 4. Familiarity was greatest in the southwestern states (52.6% of students) and declined moving north and east, with the least familiarity in the northeastern states (16.4%). Overall, familiarity with the myth was a surprising 37.2%.

### 2.4. Survey of Southern California Emergency Responders and Healthcare Professionals: Belief in the BMD Myth and Familiarity with the VD Hypothesis

We used a brief survey to assess the extent to which emergency responders and healthcare professionals in Southern California believed that babies are more dangerous (the BMD myth) and were familiar with the VD hypothesis. Three groups were represented: emergency medical technicians (EMTs)/firefighters (*N* = 19), paramedics (*N* = 34), and nurses (*N* = 22). Belief that the BMD myth was true was similar among the three groups (χ^2^ = 1.18, df = 2, *p* = 0.56, Cramer’s *V* = 0.13) and remarkably high (73.3% overall) compared to the mean level of familiarity among college students from Southern California (52.6%; see Section 2.3). Familiarity with the VD hypothesis was even higher (overall: 90.7%), but potentially differed among the three groups (χ^2^ = 7.10, df = 2, *p* = 0.029, Cramer’s *V* = 0.31; cells were sparse, but note the moderate effect size), with EMT/firefighters being highest (100%), paramedics intermediate (94.1%), and nurses lowest (77.3%).

Strong concordance existed between belief in the BMD myth and familiarity with the VD hypothesis (89.1%). However, the responses were asymmetric (McNemar test, *p* = 0.015), as 10.9% of the 55 respondents who believed babies were more dangerous had not heard the VD explanation, whereas only one of the 20 individuals who did not believe the myth (5.0%) was familiar with the VD explanation. The asymmetry suggests that the VD explanation reinforced acceptance of the BMD myth.

## 3. Discussion

In this study on the origin, spread, and social transmission of misinformation, we provide the first assessment of the origin, likely transmission, and prevalence of the claim that baby rattlesnakes are more dangerous than adults—the BMD myth. We explored the reasoning that either supported or refuted the myth, including three often-cited hypotheses and how they contributed to the spread of misinformation. The most prominent explanation to support the myth—the VD hypothesis—is the misguided notion that babies, in contrast to adults, cannot control the release of their venom and therefore dump all of it in a single bite. Limited evidence suggests that young rattlesnakes can, in fact, control venom expenditure [28]. Overwhelming evidence supports the VQ hypothesis instead, which correctly states that adults possess [21,22,23], and deliver when biting [14,20,24], much greater quantities of venom, and therefore are more dangerous.

Here, we begin by discussing the temporal dynamics of the BMD myth and the VD hypothesis. We infer that the media largely perpetuated the myth by citing non-experts with a poor understanding of rattlesnake biology and snakebite characteristics. We then address the factors that promote misinformation, the consequences of misunderstandings about rattlesnakes, and the evidence that effective messaging can dispel the myths.

### 3.1. Origin, Transmission, and Prevalence of Misinformation About the Relative Danger of Baby Rattlesnakes

When and where did the BMD myth originate, and how did it spread? From a detailed survey of newspaper stories across a 126-year period (1900–2026), we can infer that the BMD myth became prominent in the 1970s, likely driven by frequent mention in stories from California, when stories elsewhere continued the correct narrative of adults being more dangerous. The myth’s spread was clearly fueled by the widely promoted VD hypothesis, which originated from the late 1960s—we found the first mention in a 1967 story from Tennessee citing a snake hobbyist [39], Table 1—and was frequently repeated from the 1970s onward to explain the myth.

By 2000, the BMD myth and VD hypothesis, linked together, were widely proclaimed throughout North America, presumably aided by misinformation shared across the internet. Individual news stories were often carried by additional media outlets, thereby amplifying the misinformation. In one extreme case that we excluded from our analysis because we could not determine a location for its origin, the snippet “baby rattlesnake at birth has the same amount of poisonous venom as a fullgrown rattler” appeared in at least 19 newspapers on 21 May 1976, 27 additional newspapers through the end of the year, and another 16 newspapers between 1977 and 1990 (references not supplied). Beyond the newspaper stories, numerous organizations generally regarded as authoritative further amplified the information. Examples we found included popular television broadcast corporations; the Wikipedia article on *Rattlesnakes*; articles published on county, university, parenting, veterinary, companion animal, and wildlife removal websites; and a popular desert travel guide (Table 2). Much of this information remains online today. Unfortunately, we cannot assess the number of views for these sources, though the Wikipedia article for *Rattlesnakes*, which shared inaccurate information from 2005 to 2010 as the online encyclopedia was gaining attention, may have hosted hundreds of thousands of views during at least some of those years (the only reliable statistics yielded 615,411 to 346,940 user views per year from 2015 to 2025 [56], with the decline across that timespan attributed to AI-generated summaries and changing information consumption patterns [57]).

Results from the university student surveys that we conducted in 2007–2008 support our interpretation that stories originating from California were instrumental in spreading the myth. Students from the southwestern states were most familiar with the myth, and familiarity with the myth declined further east and north across the continent (Figure 4). Fortunately, the trend that began in California appeared to reverse by 2015, when the majority of stories, regardless of geographic region, increasingly provided correct information.

To bolster our understanding of the California media’s role in spreading the BMD myth and VD hypothesis, we included in our logistic regression analyses a potentially confounding variable: the context of stories. We learned that general information stories about rattlesnakes were more likely to provide accurate information than local snakebite stories, and this was driven, at least in part, by the authorities cited by the story authors. The general information stories more often cited academics (university professors), who consistently provided accurate information as subject experts, whereas the snakebite stories more often cited health professionals (e.g., physicians, veterinarians, pharmacists) and first responders (e.g., police and fire officers), who frequently supplied misinformation (Figure 3). Even so, the strong statistical interaction of geographic region and time persisted despite the significant influence of story context and authority cited.

One of the bigger surprises of our research was finding just how prevalent the BMD myth and VD hypothesis are. The survey of young adults in university courses suggested that an astonishing 37% of students in introductory biology courses were familiar with the myth. However, we were more disturbed by the remarkably high belief in the BMD myth—beyond simple familiarity—of emergency responders and health professionals in a Southern California continuing education class, at a whopping 73.3%. Many of these individuals likely learned of the myth and accepted it during their training. The asymmetric responses to our two questions from emergency responders and healthcare professionals (Section 2.4) suggest that the VD explanation reinforced acceptance of the BMD myth.

Unfortunately, we could not ascertain the precise origin of the myth, as hoped for. We have some confidence that it was not widespread in the 1960s. Herpetologist and engineer Laurence Klauber wrote the definitive book on rattlesnakes, including a chapter on myths, folklore, and tall stories. He sent out hundreds of letters of correspondence to people across the United States about rattlesnake behavior. He organized this information throughout his book, painting a vivid picture of rattlesnake myths in his time and showing that rattlesnake myths have a long-standing stature in American culture. However, he made no mention of the BMD myth in his original 1956 edition [74]. Had the myth been at all prominent by the late 1960s, we believe he would have added it to his 1972 revision [1]. His silence supports our conclusion that the myth originated quietly during the mid- to late-1960s and spread rapidly and loudly during the 1970s, with media outlets likely driving the spread.

### 3.2. The Enduring Challenge of Correcting Misinformation and Evidence of Effective Messaging

The pervasive nature of misinformation in contemporary society poses significant challenges to public understanding and informed decision-making. Dislodging inaccurate beliefs presents a serious challenge, and myths are particularly resilient. Correcting misinformation has become a prominent field of inquiry and can involve a broad range of interventions [75,76,77]. Unfortunately, refutations often inadvertently strengthen misconceptions. Studies have shown that corrections repeating misinformation can both reduce misperceptions and reinforce misinformation. Individuals often shield their pre-existing attitudes and prefer information that aligns with their existing beliefs [78]. If corrections do not offer strong alternative explanations, individuals may continue to rely on inaccurate information [79,80,81], though this still may not be sufficient [82]. We believe that emphasizing the VQ hypothesis—that the quantity of venom available and normally injected by babies is far, far less than that of adults—is important to help doubters understand why baby rattlesnakes are less dangerous. Those who serve in an education capacity, and especially those interviewed by the media, really should emphasize this. Intuitively, it seems that our effort here in explaining the origin of the myth should enhance the effectiveness of corrections.

We learned from a university professor that many students in her class refused to accept the statement read to them following the survey that baby rattlesnakes are, indeed, less dangerous than adults. She wrote, “My classes were so concerned about the topic that we actually did online research to find out if you (and I) had misled them. Thankfully, a Google [search] of your name turned up ‘envenomation expert.’ Only after we confirmed your credentials (and found some other scientific sites that presented the same argument) were they willing to accept what you had written. So yes, my classes, taking your survey, REALLY believed that the baby snakes were more toxic—even when a herpetologist and their instructor said this was a myth.”

Sadly, myths regarding rattlesnakes and other serpents abound [1,2,3,4]. Misinformation about rattlesnakes can lead to ill-informed decisions with unfortunate and sometimes serious consequences. Fear of rattlesnakes, for example, can promote hesitancy to engage in outdoor activities. News outlets often share stories in the spring, proclaiming the emergence of rattlesnakes from their winter slumber, and again during the fall, warning people that baby rattlesnake season is upon them. We have met people, for example, who say they do not like to hike in the fall because of the danger of baby rattlesnakes. This perceived excessive danger causes unnecessary fear and often provokes the needless death of rattlesnakes encountered by those who fear them. Reluctance to engage in outdoor activities promotes a sedentary lifestyle, vitamin D deficiency, and nature deficit disorder [83,84,85].

Misinformation about the relative danger of a rattlesnake can also lead to serious consequences. Some individuals might feel safer interacting with larger snakes, thinking they are safer. Misunderstanding could exacerbate fear in a snakebite victim and complicate treatment, especially if emergency responders and healthcare providers inflame the situation through their misunderstanding. Panicked patients might seek and apply drastic, unproven first-aid remedies [86], and emergency providers may succumb to pressure from patients and family members, resulting in overtreatment of the symptoms [87]. The same dynamics presumably apply to pet owners and veterinarians.

Our analyses suggest that effective messaging can succeed in dispelling even well-trenched myths. By 2015, a dramatic shift became apparent in the stories we retrieved, with news outlets increasingly getting the BMD myth right. One reason for this shift was that story authors increasingly quoted subject experts, particularly university professors. More of the stories also provided general information on rattlesnakes, likely reflecting popular interest in this charismatic group of reptiles. We found the recent trend both a surprise and a source of encouragement. Currently, a number of organizations, social media groups, and independent wildlife educators provide correct information online.

Three additional lines of evidence suggest that public understanding regarding rattlesnakes can change with effective messaging. First, the renowned American writer and folklorist J. Frank Dobie described a widespread myth from the late 1800s into the early 1900s concerning rattlesnake mothers swallowing their young [3]. He explained how Raymond Ditmars, a famous herpetologist of the Bronx Zoo in New York, successfully dispelled this myth and others over the next few decades through his lectures and writing. Second, a more recent myth, driven by sensationalistic journalism in 2008–2009, claimed that rattlesnakes were rapidly evolving supertoxic venoms, leading to increasingly more severe human envenomations. The myth promptly disappeared from media coverage after a paper published in 2010 thoroughly debunked the claim [88]. Third, a handful of survey studies reveal that experiential learning involving snakes—specifically, holding and/or keeping non-venomous snakes, observing any snakes in nature, and even watching educational videos—can enhance positive attitudes, though demographic factors such as gender and religion can influence effectiveness, and long-term change remains unstudied [89,90,91,92,93,94]. Collectively, these examples demonstrate that both factual understanding and public opinion can indeed be modified through education and advocacy, especially when it originates from professionals in the field.

## 4. Conclusions

In this study, we undertook three primary efforts to explore the origin, transmission, and prevalence of the BMD myth. We examined historical newspaper accounts across North America; we surveyed university student familiarity with the myth and the most frequent explanation for it, the erroneous VD hypothesis, in classrooms across the United States; and we conducted a small survey of Southern California emergency responders and health professionals. We found that the myth likely originated in the 1960s and gained increasing traction during the 1970s, largely fueled by media coverage in California. After becoming remarkably prevalent across the continent in the 2000s, journalists increasingly cited relevant experts and got the story right from about 2015 onward.

These findings underscore the need for effective messaging—including through the same media that perpetuates misconceptions—which can help police and fire officers, emergency responders, poison control centers, health professionals, companion animal organizations, wildlife agency staff, and the general public gain a more accurate understanding of rattlesnake behavior and snakebites. We hope that dissemination of these findings will inspire news stories aimed at reducing misinformation and unwarranted fears of rattlesnakes. Changing attitudes toward baby rattlesnakes will help people more safely coexist with these venomous creatures.

## 5. Materials and Methods

### 5.1. Assessment of Newspaper Stories

To locate articles on venom use by baby rattlesnakes, we conducted online searches of newspaper accounts using the Newspapers.com archive, Google News, and Google. The key words most often used together included baby, adult, rattlesnake, control, venom, and dangerous. However, we also interchanged additional words, including small, juvenile, neonate, larger, bigger, poison, and bite. The most efficient searches combined “baby rattlesnake” and “more dangerous.” We screened all candidate stories for the period 1900 to 2025. When a search retrieved multiple versions of the same story, we sought the earliest published and disregarded the others.

For data extraction, we checked each story to assess whether mention of the relative danger of baby rattlesnakes was factually correct (see Supplementary Materials Table S1). We also recorded explanations and factual correctness for why baby and adult rattlesnakes differ in relative danger, including their capacity to control venom release, the amount of venom injected, the composition of their venom, and other reasoning. We further determined whether an authority was cited and, if so, the authority’s general employment category. Occupation categories included: (1) university professor; (2) health professional (e.g., physician, veterinarian, pharmacist, medical student); (3) fire or police officer; (4) government agency staff member (e.g., animal control officer, park ranger or naturalist, fish and wildlife service warden); (5) museum or zoo staff member; (6) business owner (usually involving snakes) or hobbyist; (7) the story author; or (8) other (e.g., bite victim, non-governmental agency staff, life insurance agent, unknown). Because exploratory analyses revealed a stark contrast in factuality between stories originating from California and elsewhere, we dichotomized the geographic source of each story as California or other. To control for the possibility that stories describing local snakebite cases might rely on less- or better-informed authorities than general information stories about rattlesnakes, with California possibly biased toward more or fewer snakebite stories, we further dichotomized the context of each story as bite or general information.

### 5.2. Survey of University Students Across the United States

To assess regional familiarity with the BMD myth and VD hypothesis, we identified General Biology instructors at numerous colleges and universities and one high school willing to administer a brief survey. We asked the instructor to read the following statement out loud at the beginning of a class session: “In cooperation with Dr. William Hayes, a researcher at Loma Linda University, California, I am conducting a very simple single-question survey. The results will be published in a professional journal. The question is: Have you heard that baby rattlesnakes are more dangerous than adults because they have not learned to control the amount of venom they inject when biting, and therefore inject more? If you have heard this before, please raise your hand.” The instructor counted the number of hands raised and the total number of students present before reading out loud the correct facts about the relative danger of baby rattlesnakes, their capacity to modulate venom expenditure, and why a factual understanding is beneficial. The surveys were conducted from September 2008 to March 2009. After compiling the survey results, we aggregated the percentage familiar with the myth in each classroom according to six geographic regions: the northwest, southwest, north-central, south-central, northeast, and southeast states (see Supplementary Materials Table S2).

### 5.3. Survey of Southern California Emergency Responders and Health Professionals

In April 2009, we gave a safety lecture on rattlesnakes to a group of emergency responders and health professionals at a Riverside County Emergency Medical Services continuing education program in Riverside, California. At the start of the lecture, we handed out a piece of paper to all participants, asking “Do you believe that baby rattlesnakes are more dangerous than adults?” (the BMD myth) and “Have you heard that baby rattlesnakes are more dangerous than adults because they have not learned to control the amount of venom they inject when biting, and therefore inject more?” (the VD hypothesis). We also asked which of the three groups each participant was a member of: (1) student; (2) EMT/firefighter, (3) paramedic, (4) nurse, or (5) physician. We had just two responses from students and none from physicians, so these two categories were omitted from analyses.

### 5.4. Statistical Analyses

We conducted all analyses using IBM SPSS Statistics for Windows, Version 23.0 (IBM Corp., Armonk, NY, USA), with alpha set at 0.05.

For the newspaper analyses, we subjected the correctness of BMD stories and of stories mentioning the VD hypothesis to binary logistic regression analyses. We included three predictors: year of publication (1900–2025), region (California versus elsewhere), and story context (general information or snakebite). We used AIC to assess alternative models with all three main effects and various combinations of two-way and three-way interactions. We then conducted chi-square analyses to compare (1) the proportions of general information and snakebite stories (context) across the four time periods with varying story-telling accuracy (1900–1969, 1970–1999, 2000–2014, 2015–2025) and (2) the factuality of stories across all time periods citing authorities within the different employment categories. Cell counts for the eight employment categories were adequate for the BMD stories but sparse for stories mentioning the VD hypothesis. For the latter, we collapsed four employment categories having nine or fewer stories, leaving four categories for analysis. We also used chi-square tests to assess, separately, the proportions of academics and health professionals cited during different time periods in stories mentioning the BMD myth (time periods 2–4) and VD hypothesis (time periods 3 and 4). For the chi-square tests, we computed Cramer’s *V* (when more than four cells) or phi (φ, when four cells) as a measure of effect size, with small, medium, and large effects loosely corresponding to 0.1, 0.3, and 0.5, respectively [95].

For the university student surveys, we subjected the mean values of familiarity per classroom to a one-way analysis of variance (ANOVA), with the six geographic regions treated as an independent (between-subjects) variable. We computed eta-squared (η^2^) as a measure of effect size, with small, medium, and large values loosely corresponding to 0.01, 0.06, and 0.14 [95].

For the Southern California emergency responders and health professionals survey, we again relied on chi-square tests and Cramer’s *V* to compare belief in the BMD myth and familiarity with the VD hypothesis among the three occupation groups. We also used a McNemar test to assess whether the proportions of discordant responses (mismatched yes/no answers) differed between belief in the BMD myth and familiarity with the VD hypothesis.

## Figures and Tables

**Table 2:** Examples of organizations that have amplified the myths that baby rattlesnakes are more dangerous than adults and/or cannot control venom expenditure when biting. Some website dates were determined via the Internet Archive [57].

Organization and Media	Year(s)	Source
Doberman Rescue of New Mexico: *Rattlesnake bites* (website article)	2026	[58]
Vet Near Me, Boise, Idaho: *Rattlesnake bite risks in dogs: Prevention and treatment* (website article)	2025–2026	[59]
County of Los Angeles, California, Animal Care and Control: *Managing rattlesnake problems* (website article)	2025–2026	[60]
Natural Parenting Center: *Are baby rattlesnakes more dangerous?* (website article)	2025–2026	[61]
The Tucson Dog Magazine, Tucson, Arizona: *Snake and toad avoidance training might just* *save your pet’s life* (website article)	2024–2026	[62]
Pepperdine University, Malibu, California: *Campus wildlife* (website article)	2023–2026	[63]
AAAC Wildlife Removal (17 states): *Baby rattlesnakes: Tiny but dangerous? What you need* *to know* (website article)	2022–2026	[64]
Adobe Veterinary Center, Tucson, Arizona: *Rattlers on the move* (website article)	2014–2026	[65]
County of Los Angeles, California, Public Health: *Rattlesnake season returns* (website article)	2008–2026	[66]
Discovery Channel: *Naked and afraid XL*, season 5, episode 6 (television series)	2019	[67]
Idaho Geocachers: *Idaho rattlesnakes* (website article)	2004–2017	[68]
Pinnacle Peak Animal Hospital, Scottsdale, Arizona: *Rattlesnake bites—A guide to prevention* *and treatment* (website article)	2004–2011	[69]
Pepperdine University, Malibu, California: *Rattlesnakes don’t make good pets* (website article)	2007–2010	[70]
Wikipedia: *Rattlesnake* (website article)	2005–2010	[71]
National Geographic Channel: *The Animal Extractors* (television series)	2007	[72]
American Automobile Association: *Desert Areas Guidebook* (book)	1996	[73]

## Data Availability

The original contributions presented in this study are included in the article/Supplementary Material. Further inquiries can be directed to the corresponding author.

## Supplementary Materials

The following supporting information can be downloaded at https://www.mdpi.com/article/10.3390/toxins18030144/s1, Table S1: News media stories that mentioned the relative danger and/or venom use by baby rattlesnakes [96,97,98,99,100,101,102,103,104,105,106,107,108,109,110,111,112,113,114,115,116,117,118,119,120,121,122,123,124,125,126,127,128,129,130,131,132,133,134,135,136,137,138,139,140,141,142,143,144,145,146,147,148,149,150,151,152,153,154,155,156,157,158,159,160,161,162,163,164,165,166,167,168,169,170,171,172,173,174,175,176,177,178,179,180,181,182,183,184,185,186,187,188,189,190,191,192,193,194,195,196,197,198,199,200,201,202,203,204,205,206,207,208,209,210]. Table S2: Responses of United States university students in introductory biology courses, by region, to a survey question about familiarity with the babies-more-dangerous myth and the venom-dump hypothesis.

## Abbreviations

The following abbreviations are used in this manuscript:

BMD	Babies-more-dangerous (myth)
VD	Venom-dump (hypothesis)
