# Supplementary material for: Are Baby Rattlesnakes More Dangerous than Adults? Origin, Transmission, and Prevalence of a Media-Driven Myth, with Evidence of Effective Messaging to Dispel It
Source: Toxins (Basel). 2026 Mar 14;18(3):144. doi: 10.3390/toxins18030144 (PMC13030322; doi:10.3390/toxins18030144)
Supplement: Supplementary file 1 [file toxins-18-00144-s001.zip › SUPPLEMENTAL TABLE S1 PROOFS W WKH EDITS.pdf]

Supplemental Table S1. News media stories from the United States and Canada that mentioned the relative danger of and/or venom use by baby rattlesnakes. We located via searches of Newspapers.com, Google News, and Google searches during the period 1900–2025 (N = 130; see Methods in manuscript). Comments are direct quotes from the stories, which may or may not have accurately represented the authority's remarks when cited. Context of the story was either general interest (non-bite) or one or more regional snakebite cases (bite). Authorities cited in the story (Auth) were categorized as AC = Academic (College/University professor); AS = Agency staff (Animal Services, Fish and Game, Park ranger, Naturalist); AU = author of story; BH = Business owner/employee, hobbyist, snake remover; HP = Health professional; FP = Fire/Police officer; OT = Other; ZM = Zoo/Museum staff. Factuality (true or false) was assessed for mention of the relative danger of babies (babies-more-dangerous [BMD] myth), the venom-dump (VD) hypothesis (which is incorrect), the venom-quantity (VQ) hypothesis (which is correct), and venom-toxicity (VT) hypothesis (which has mixed evidence, and therefore no assessment of factuality was made). Sources (numbers in brackets) correspond to the list of References in the manuscript.

Year	State/ Province	Comment (authority, if cited)	Context	Auth	Hypothesis				Source
					BMD	VD	VQ	VT	
2025	CA	"While they're smaller in size, a bite from a baby rattlesnake can be just as dangerous if treatment isn't swift. Youngsters release less venom but their poison can be more potent, according to Taylor." <Authority cited: Emily Taylor, biological sciences professor at Cal Poly in San Luis Obispo>	Non-bite	AC	True		True	X	[96]
2025	SC	"Some fear baby rattlesnake bites are more potent than adults because they cannot control their venom. This is a myth." <Authority cited: Asclepius Snakebite Foundation>	Non-bite	AC	True	True			[97]
2025	WA	"…much of what we think we know about baby rattlesnakes is a myth…There's two big ones that I hear over and over – that their venom is more toxic than that of adult snakes and that they can't control the amount of venom they release," he said. "Neither one of these is true. Their venom is not higher in toxicity, and yes, they do regulate how much venom they inject." <Authority cited: Charles Peterson, professor emeritus of herpetology at Idaho State University>	Non-bite	AC	True	True		X	[98]
2025	GA	"Contrary to popular belief, the bite of a baby rattlesnake is almost always far less serious than the bite of a larger adult rattlesnake. The notion that baby rattlesnakes cannot control the quantity of venom injected is a myth that has been disproven multiple times through well-designed studies" <Authority cited: Ascelpius Snakebite Foundation>	Non-bite	AC	True	True			[99]
2025	CO	"The myth is baby rattlesnakes are more dangerous to people because they can't control the amount of venom in their bites. The truth is a baby rattlesnake has far less venom than an adult." <Authority cited: Project Rattlecam>	Non-bite	AC	True	True	True		[100]
2025	CA	"She also debunked the widespread rumor that bites from baby rattlesnakes are more dangerous than bites from adult snakes. "That's not true in any way, shape or form…Babies can in fact control their venom." <Authority cited: Emily Taylor, professor of biological	Non-bite	AC	True	True			[101]

		sciences at Cal Poly>								
2024	PA	“Are baby rattlesnakes more dangerous than adults? No! Baby rattlesnakes actually have less venom than their parents, but they are still potentially dangerous from the day they are born. Both babies and adults can meter their venom. That means they can choose to deliver a warning bite with no venom at all.” <Authority cited: None>	Non-bite	AU	True	True	True		[102]
2024	CA	“Baby rattlesnakes are not more dangerous than adults. They have tiny venom glands. Adults, having much larger venom glands, possess much more venom and are therefore more dangerous.” <Authority cited: None>	Non-bite	AU	True		True		[103]
2024	AL	“Some people will claim that baby snakes are more dangerous than adults…this idea is false. Baby snakes can control how much venom is injected, just like adults, and their venom is not more concentrated.” <Authority cited: None>	Non-bite	AU	True	True		X	[104]
2024	AZ	“…baby rattlesnakes aren't more dangerous than adults. A baby rattlesnake's venom is just a tiny fraction of that produced by an adult.” <Authority cited: Cale Morris of the Phoenix Herpetological Sanctuary>	Non-bite	ZM	True		True		[105]
2023	CA	“…says it is not true that the larger the rattlesnake, the more venom it will deliver when it injects.” <Authority cited: Laura Patterson, Amphibian and Reptile Conservation Coordinator with the California Department of Fish and Wildlife>	Non-bite	AS	False				[106]
2023	ID	“"Baby rattlesnakes are not more dangerous than adults, they can control their venom unlike the popular myth.” <Authority cited: Brett Parker of Hill Country Snake Removal>	Non-bite	BH	True	True			[107]
2023	BC, Canada	“Bites from baby rattlesnakes are particularly dangerous because, unlike their adult counterparts, they cannot yet control how much venom they inject.” <Authority cited: None>	Non-bite	AU	False	False			[108]
2022	CA	“There is a common myth that [rattlesnake] babies are more dangerous. The saying is that babies can't control the venom and release more of it. There is no truth to that. The babies can control the amount of venom just like the adults can.” <Authority cited: Dr. William Hayes, professor of biology for Loma Linda University>	Bite	AC	True	True			[109]
2022	AZ	“…while the snake babies are venomous, they are no more dangerous than their mothers. ‘It's a myth, a really popular myth that baby rattlesnakes are more dangerous…Baby rattlesnakes have just as much control over venom expenditure as adults, but they are not as bad because they don't have as much venom to give.’" <Authority cited: Bryan Hughes, Rattlesnake Solutions owner>	Non-bite	BH	True	True	True		[110]
2022	CA	“It’s a myth that these babies are more dangerous than adults, due to delivering more venom in bites, he says. “It’s not true. Baby rattlesnakes	Non-bite	BH	True	True			[111]

		have just as much control over venom expenditure as adults…” <Authority cited: Bryan Hughes, owner of Rattlesnake Solutions>							
2022	AZ	“Baby snakes are much less dangerous than adult rattlesnakes for numerous reasons, contrary to myths.” <Authority cited: Cale Morris, Phoenix Herpetological Society's venom manager>	Non-bite	ZM	True				[112]
2021	FL	“It's common knowledge that juvenile rattlesnakes can't control their venom and are therefore more dangerous than adults. It's also completely untrue. Baby rattlers and other venomous snakes are born with the ability to regulate exactly how much venom is administered in a bite. They have to be, because they have much smaller venom sacs and can't afford to waste what little they've got.” <Authority cited: None>	Non-bite	AU	True	True			[113]
2021	AZ	“Baby rattlesnakes are not more dangerous than adult rattlesnakes…When it strikes you it will inject venom but have no control of it.’ However, young rattlesnakes have a venom gland the size of a sewing pin so they can only inject a nominal amount of venom.” <Russ Johnson, President of the Phoenix Herpetological Society>	Non-bite	ZM	True	False	True		[114]
2020	TX	“Baby rattlesnakes are not more dangerous than adult rattlesnakes…Babies actually ‘have less venom to inject when they bite…’” <Authority cited: Brian Todd, University of California Davis conversation biology professor>	Non-bite	AC	True		True		[115]
2020	CA	“Bites from baby or adult rattlesnakes are both dangerous and medical emergencies…She dispelled the myth that baby rattlesnake bites are more dangerous with the thought they cannot control their venom. ‘That is absolutely not true…Bites from adults are far worse than babies.’” <Authority cited: Emily Taylor, biology professor at Cal Poly>	Non-bite	AC	True	True			[116]
2019	Alberta, Canada	“And one particular rattler you don’t want to get bit by are the baby rattlers. ‘The adult snakes have a little bit more control of how much venom they can inject into someone, where with the young snakes, they haven’t learned that as well and so they may give you a full dose.’” <Authority cited: Mary Drut, park interpreter>	Bite	AS	False	False			[117]
2019	VA	“If you have a baby snake and you go pick that up and it bites you, it's going to dump its entire venom reserve into you. An adult snake can give you a dry bite, meaning they can bite you and inject no venom at all.” <Authority cited: Brian Fiske with ACME Animal Control>	Non-bite	AS	False	False			[118]
2019	CA	“It may be true that juvenile rattlesnakes can be more likely to strike and less able to control how much venom they release in a single bite. Their venom also can be, drop for drop, more dangerous because of the different prey that juvenile snakes eat. But it’s still more dangerous to receive a bite from an adult rattlesnake. Adults possess much more venom, making the bites more dangerous.” <Authority cited: None>	Non-bite	AU	True	False	True	X	[119]

2019	CA	“Though young rattlers are said to be unable to control their venom (though that’s disputed by some), ‘bigger snakes have bigger venom sacs, and adults can deliver far greater volumes of venom than babies.” <Authority cited: Greg Pauly, herpetologist, Los Angeles Natural History Museum>	Non-bite	ZM	True	False	True		[120]
2018	CA	“…when they bite, they release all their venom, unlike mature snakes that control the amount they release.” <Authority cited: Rick Dickinson, Fire Chief>	Bite	FP	False	False			[121]
2017	SD	“Baby rattlesnakes are a [sic] little mimics of their parents, they have full control over their venom delivery system.” <Authority cited: Terry Phillip, Reptile Gardens curator of reptiles>	Non-bite	ZM	True	True			[122]
2017	CA	“Myth: The worst snakebites are those from baby snakes. That thinking began in the California in the 1970s and spread in the 1990s…a baby rattlesnake has much less venom than adults…so the amount of toxin delivered won’t be very much compared to an adult snake. Immature snakes haven’t learned to control the amount of venom they release.” <William Hayes, a rattlesnake researcher and biologist at Loma Linda University – misquoted on VD hypothesis>	Bites	AC	True	False	True		[55]
2017	TX	“"Adult rattle snakes have a little bit more control, so a baby rattle snake may eject a person or an animal that they come into contact with a lot of venom where an adult rattle snake may not do so.” <Authority cited: Katrena Mitchell, Administrator for the City of Wichita Falls Animal Services>	Non-bite	AS	False	False			[40]
2017	AZ	“A father, attending with his son, said baby scorpions are more dangerous than adults since they can't control their venom—a myth that's also commonly attributed to snakes…he said he'd rather be bitten by a baby rattlesnake than the ginormous adult any day, since the babies don't have nearly as much venom.” <Authority cited: Mark Paulat, park ranger>	Non-bite	AS	True	True	True		[123]
2017	CA	“They warned her that baby rattlers are even more dangerous than adults because they can't control their venom.” <Authority cited: Los Angeles County Fire>	Bite	FP	False	False			[124]
2015	CA	“Some snake experts say baby rattlesnakes can deliver a more-potent bite than an adult because they can’t control the dose they deliver, but…no studies support that theory.” <Authority cited: Dr. Richard Clark, director of the Division of Medical Toxicology at UC San Diego Health System>	Bite	HP	True	True			[125]
2013	Alberta, Canada	“The younger snakes are actually more dangerous than the adults because the adults have more control over how they disperse their venom.” <Authority cited: Crown Prosecutor Christian Lim> [NOTE:	Non-bite	OT	False	False			[41]

		"Lim said in court"]							
2013	CA	"Are baby snakes more dangerous? The truth behind this is slim to none. I'd rather get bitten by a baby than an adult. Adults' fangs are longer, and they can inject venom deeper. Also, the amount of venom in their glands is far greater than what a juvenile possesses." <Authority cited: Magee of Magee's Orange County Snake Removal>	Non-bite	BH	True	True	True		[126]
2013	AZ	"'The venom is not worse; they cannot deliver more venom than an adult…It would always be worse to be bitten by an adult.'" <Authority cited: Nate Deason, serpent curator at the Phoenix Herpetological Society>	Non-bite	ZM	True		True		[127]
2012	CA	"…baby rattlers often expel more venom than their adult counterparts. 'They don't know when to stop biting,'" <Authority cited: Sgt. Chris Chapman of the East Bay Regional Parks Police>	Bite	FP	False	False			[128]
2012	AZ	"The babies…bite is just as deadly as the big ones…The babies have just as much venom as adult rattlers but they can be more dangerous because they bite more than once and it feels more like a pinch instead of an actual bite." <Authority cited: Daniel Marchand with Phoenix Herpetological Society>	Non-bite	ZM	False		False		[129]
2011	CA	"Baby rattlers may be even more dangerous than adults…An adult will try to save its venom to immobilize its prey, and may not inject any when defensively striking a human. A juvenile, though, doesn't know yet how to moderate its venom 'dose,' so it could give you the full wallop." <Authority cited: Barbara Norton, supervising ranger at Laguna Coast Wilderness Park>	Bite	AS	False	False			[42]
2011	CA	"Young rattlesnakes can be more dangerous as they have not developed the ability to control the flow of venom, meaning all the venom is released during a bite…While an adult rattlesnake can control the flow of the venom it releases, a juvenile will release all the venom it has, which ultimately requires a larger dose of antivenin to treat." <Authority cited: Dr. Robbie Cohen, Chief Medical Officer and ER Physician>	Bite	HP	False	False			[43]
2011	CA	"…young rattlesnakes…are the most dangerous ones since they can't control their venom output and will release all of it in you, unlike a grown rattler, which will either give you a warning bite or only release a portion of its venom." <Authority cited: None>	Non-bite	AU	False	False			[130]
2011	PA	"baby rattlesnakes are much more dangerous—and venomous—than adult rattlesnakes." <Authority cited: Brandon Fournier, a trained snake handler>	Bite	BH	False	False		X	[131]
2011	CA	"Baby snakes are more dangerous than adults because they don't have control over how much venom they inject when biting." <Authority cited: None>	Non-bite	AU	False	False			[132]

2011	UT	"…do not touch a snake, especially baby rattlesnakes, because unlike adult rattlesnakes, they can't control their venom. That makes them even more dangerous." <Authority cited: James Dix of Reptile Rescue Service>	Bite	BH	False	False			[133]
2010	CA	"Baby rattlesnakes can be more dangerous than fully grown rattlesnakes because they might unleash all their venom when they bite." <Authority cited: Ryan Drabek, interim director of OC Animal Care>	Bite	AS	False	False			[134]
2010	CA	"…baby rattlesnakes, in addition to not being able to control the amount of venom they inject, tend to strike more often because their rattles aren't effective tools for warding off predators. <Authority cited: Paul Collins, vertebrate zoologist, Santa Barbara Museum of Natural History>	Bite	ZM	False	False			[135]
2009	CA	"Myth: A baby rattler's bite is more dangerous than an adult's. Reality: This is based on the fact that, drop for drop, a baby rattlesnake's venom can be more toxic. However, an adult rattler has much more venom than a baby. So a bite from an adult rattler is almost always worse than one from a baby." <Authority cited: Emily Taylor, Cal Poly assistant biology professor>	Non-bite	AC	True		True	X	[136]
2009	UT	"…baby rattlesnakes are potentially more dangerous than larger ones… adult snakes only release venom 75 percent of the time. 'Babies its one-hundred percent [envenomation]. You get bit by a baby, it doesn't save its venom for its prey. It doesn't know any better. It will invenomate [sic] you every time it strikes, and they can do that several times,' he said." <Authority cited: James Dix, founder of Reptile Rescue>	Non-bite	BH	False	False			[44]
2009	FL	"Typically speaking, smaller rattlesnakes are less dangerous than larger rattlesnakes." <Authority cited: Terry Ferrell, Stetson University Biology Professor>	Bite	AC	True				[137]
2009	ID	"'The juvenile rattlesnakes do have more concentrated venom ... but bigger snakes inject a lot more venom,' Huntington said." <Authority cited: Dr. Thomas Huntington, director of Minimally Invasive Surgery at Saint Luke's Boise Medical Center>	Bite	HP	True		True	X	[138]
2009	TX	"Baby venomous snakes are far more dangerous than adults. A baby snake will inject all its poison in a single bite; an adult will inject only a small portion of its poison." <Authority cited: None>	Non-bite	AU	False	False			[139]
2009	AZ	"Younger rattlesnakes also have less control over their venom. An adult rattlesnake can decide how much venom it wants to inject into you, but baby rattlesnakes, they can't control their venom, said McCluskey." <Authority cited: Jerry McCluskey, station pest controller>	Non-bite	AS	False	False			[140]
2008	CA	"Bites from baby rattlesnakes often are worse than those from adult snakes…They can't control their venom release like an adult rattlesnake	Bite	HP	False	False			[141]

		can, so you get much more of a dose of venom.” <Authority cited: Greg Kennedy, emergency medical services coordinator and clinical educator for the county fire district>							
2008	CO	“Adult rattlesnakes can choose to inject or not inject venom when biting. Baby snakes don't have as much control. This is why an animal or human can be bitten by a large rattlesnake and suffer little consequence. There may be very little or no venom delivered during the bite.” <Authority cited: None>	Non-bite	AU	False	False			[142]
2008	TN	“Of course, by that time they’d almost lost the preacher (those younger snakes really use all their venom the first time) and the preacher sure was glad the Lord had summoned the doctors to help in the 10 days that followed in intensive care.” < Authority cited: None>	Bite	AU	False	False			[143]
2008	CA	“Baby rattlers are the most dangerous as they are agile and nervous, prone to strike out of fear, and the concentrated venom is 13 times more potent than an adult's.” <Authority cited: None>	Bite	AU	False			X	[144]
2008	VA	“...a juvenile rattlesnake is not usually large enough to deliver enough venom to be lethal...There's an old wives' tale that says a baby rattlesnake bite is worse than an adult bite, but that's just not true.” <Authority cited: Bob Myers, director, American International Rattlesnake Museum, New Mexico>	Bite	ZM	True	True	True		[145]
2007	CA	“…a bite from a baby rattler delivers a great deal more venom, because they don't have the control that adult snakes do.” <Authority cited: Bo Slyapich, “rattlesnake wrangler”>	Bite	BH	False	False			[146]
2007	AZ	“...baby rattlesnakes have no control over their venom release, so their victims usually receive a full dose.” <Authority cited: Russ Johnson, President, Phoenix Herpetological Society>	Non-bite	ZM	False	False			[147]
2007	MT	“All rattlesnakes can control how much venom they inject…the rumor is that younger snakes can’t control it as well.” <Authority cited: Allison Begley, Department of Fish, Wildlife and Parks native species biologist>	Non-bite	AS	True	True			[148]
2007	TN	“Theoretically, the larger the snake, the more venom that he can inject in you when he bites...big or little, they have some control over the amount of venom they produce.” <Authority cited: Ken Childress, Bays Mountain Park naturalist”>	Non-bite	AS	True	True	True		[149]
2007	TX	“…the smaller rattlesnakes are the worst ones because they have no control over its venom...” <Authority cited: Travis Tate, snakebite victim>	Bite	OT	False	False			[150]
2007	TX	“Another misconception is that the smallest snakes are the worst one [sic] to be bitten by...the larger the snake, the more venom they carry and the worse they bite...” <Authority cited: Dr. David Greer, Physician>	Non-bite	HP	True		True		[151]
2007	CA	“…baby snakes are notorious for releasing all their venom on the victim	Bite	HP	False	False			[152]

		at once." <Authority cited: pediatric intensive care physician>							
2006	CA	“Infants aren’t more deadly than adults.” <Authority cited: Inland Valley Humane Society>	Non-bite	AS	True				[153]
2006	CA	“The commonly held belief that a baby snake's bite is more lethal than an adult's is just legend…” <Authority cited: Robert Applegate, retired firefighter/snake breeder>	Non-bite	BH	True				[154]
2005	CO	“Generally, the bigger the snake, the worse the bite. An exception is that baby rattlers can inflict severe bites – not, as legend has it, because they haven’t learned to control the amount of poison, but because their venom is actually more poisonous.” <Authority cited: None>	Bite	AU	False	True		X	[155]
2005	AZ	“Baby snakes can be as dangerous as adult rattlers because they haven't learned to conserve their venom.” <Authority cited: Craig Ivanyi, herpetologist, Arizona-Sonora Desert Museum>	Bite	ZM	False	False			[156]
2005	CO	“But the baby snake can't inject as much venom as an adult.” <Authority cited: Jennifer Clarke-Mackessy, professor, Northern Colorado University>	Bite	AC	True		True		[157]
2004	CA	“Smaller, younger snakes release more venom than older and larger snakes because they have not yet learned to control the amount of venom put out.” <Authority cited: None>	Non-bite	AU	False	False			[158]
2004	CA	“Young snakes are much more dangerous than older snakes because they don't know how to control their venom...they don't know how to regulate...you'll get everything they have.” <Authority cited: Robert Real, field supervisor, West Valley Mosquito and Vector Control District>	Bite	AS	False	False			[159]
2004	CA	“While it is true that the venom of baby snakes is more concentrated and therefore potentially more lethal, the volume of venom in these babies is much smaller than in the adults…Even though the baby rattlers release their entire amount of venom with each bite, the total dose received is often less than their adult counterparts. <Authority cited: None>	Non-bite	AU	True	False	True	X	[45]
2004	CA	“…adult rattlers can control the amount of venom they inject, while babies are more likely to inject all their venom when they bite.” <citing publication, “Rattlesnake Bite,” The Los Angeles Regional Drug and Poison Information Center>	Non-bite	HP	False	False			[160]
2004	NM	“Some people may have heard that a young rattlesnake is more dangerous than an adult because it releases more venom than an adult when it strikes.” <Authority cited: None>	Non-bite	AU	False	False			[161]
2003	CA	“While the adult may preserve its venom when it strikes, a baby rattlesnake tends to unleash all of its venom because it is inexperienced.” <Authority cited: Joel Robinson, Nature Conservancy Guide>	Non-bite	OT	False	False			[162]
2003	CA	“The little guys will dump everything they have in their victim, because	Bite	HP	False	False			[163]

		they haven't learned any control.” <Authority cited: Richard Rosebrock, veterinarian>	(dogs)						
2002	CA	“Small rattlesnakes are more dangerous than the larger ones because they don’t gauge how much to inject the way larger snakes might.” <Authority cited: Melinda Head, a member of Marshall Hospital’s emergency department administration>	Non-bite	HP	False	False			[164]
2002	CA	“…a baby rattler, which is considered more dangerous than adult snakes because it doesn't know how to control the release of its venom.” <Authority cited: None>	Non-bite	AU	False	False			[165]
2002	OR	“…unlike adults, young rattlesnakes can't control the amount of venom they inject…” <Authority cited: Mary Esther Hart-Brown, owner of Hart's Reptile World in Canby>	Bite	BH	False	False			[166]
2001	CA	“…because they also do not regulate venom, bites from babies can be much more serious.” <Authority cited: Ralph Waterhouse, director of Chaffee Zoological Gardens in Fresno>	Bite	ZM	False	False			[167]
2000	CA	“Juvenile rattlesnakes, like adult rattlesnakes, pose the same danger if encountered and threatened. But because of the young rattlesnakes’ inexperience with the amount of venom they inject into their prey, they can be deadlier, experts warn.” <Authority cited: None>	Non-bite	AU	False	False			[168]
2000	CA	“...inexperienced younger rattlesnakes cannot control the amount of venom they release...have a tendency to attack unprovoked and then cling to their targets until they are physically removed...release all the venom they have.” <Authority cited: Mark Vincent, supervisor at Ed Davis Park, Towsley Canyon>	Non-bite	AS	False	False			[169]
2000	FL	“…the [baby's] bite is more dangerous than that of the adult rattler. Just because it's juvenile doesn't mean it has less potency—it has more potency...about four to ten times.” <Authority cited: Lynn Fenimore, Native Village wildlife educator>	Bite	ZM	False	False			[170]
2000	TX	“Smaller snakes are more likely to inject more venom because they have not learned to control their bite...” <Authority cited: Rafael De La Garza, Captain, Victoria Fire Department and EMS>	Bite	FP	False	False			[171]
1999	CA	“The baby rattlesnakes are more dangerous than the adults because they cannot control the venom they excrete. The adults can bite a person without releasing venom, unlike the babies.” <Authority cited: Eva Montes, animal control aid, Los Angeles County Animal Care and Control in Castaic>	Non-bite	AS	False	False			[172]
1999	CO	“…a baby, but they pack just as much venom in their bites as an adult snake, experts say.” <Authority cited: None>	Bite	AU	False				[173]
1998	CA	“Baby rattlesnake bites are more severe since babies can't control the amount of venom they release...An adult snake releases smaller amounts,	Bite	AS	False	False			[174]

		or no venom at all.” <Authority cited: Lt. Marie Hulett of the Orange County Animal Shelter>							
1998	CA	“Young serpents tend to inject most of their venom when they bite whereas adult snakes have more control over the discharge of poison” <Authority cited: Richard Dyatt, Fire Captain>	Bite	FP	False	False			[175]
1998	MT	“Some people consider young snakes to possibly be more dangerous than adult snakes.” <Authority cited: None>	Bite	AU	False				[176]
1997	CA	“Unlike adult rattlers, which regulate the venom dosage depending on the size of the prey, baby rattlers often shoot their entire venom wad when they bite.” <Authority cited: None>	Bite	AU	False	False			[177]
1997	CA	“A baby rattler, some experts believe, is more dangerous than a full-grown adult rattler...baby rattlers haven't yet mastered the art of injecting only the amount of venom needed to paralyze their prey, as adult rattlers do. They lock on and empty their entire reservoir of venom.” <Authority cited: None>	Non-bite	AU	False	False			[178]
1996	UT	“All else being equal, the bigger the snake the bigger volume" of venom that can be injected.” <Authority cited: Jack Sites, professor, Brigham Young University>	Non-bite	AC	True		True		[179]
1995	CA	“The smaller snakes can pack the bigger bites. The young rattlers ‘can't control the amount of venom they inject...sometimes adults can strike but not envenomate very much.’” <Authority cited: Robert Dimand, Director of Pediatric Intensive Care Unit at UC Davis Medical Center>	Bite	HP	False	False			[180]
1995	CA	“The baby snakes are also more dangerous than the adults. The adult snakes inject only enough venom into their prey as necessary and often do not inject venom into a person. A baby snake doesn't make that judgment and injects all of its venom.” <Authority cited: Bill Krumbein, Ranger, Annadel State Park>	Bite	AS	False	False			[46]
1994	CA	“…baby rattlesnakes...are not able to control the amount of poison injected.” <Authority cited: None>	Non-bite	AU	False	False			[181]
1992	UT	“The larger the snake, the more dangerous the bite.” <Authority cited: None>	Bite	AU	True				[182]
1992	CA	“…baby rattlesnakes can be more dangerous than adults, because they don't yet control the amount of venom released in a bite.” <Authority cited: Susan Kim, Clinical Pharmacist for Poison Control Center>	Bite	HP	False	False			[183]
1991	TX	“…the bigger the snake, the more venom it has.” <Authority cited: Thomas Glass, Jr., physician>	Bite	HP	True		True		[184]
1991	TX	“…there are also a number of misconceptions…the bite of a small rattlesnake is more dangerous than that of a large…possibly comes from the fact that there are more smaller snakes and therefore possibly more people are bit by smaller snakes.” <Authority cited: Bill Lamar, adjunct	Non-bite	AC	True				[185]

		professor at the University of Texas at Tyler>							
1987	NY	"…young rattlesnakes are more dangerous, but not because they are quicker. Older snakes develop sphincter control and save some venom for the next bite; young ones give you the whole shot right off - more venom." <Authority cited: None>	Non-bite	AU	False	False			[47]
1987	CA	"Being bit by a baby rattlesnake is worse than being bit by an adult because babies don't control the amount of venom they put out the way adults do...A baby just goes for it." <Authority cited: Debbie Fulbright, animal services officer>	Bite	AS	False	False			[186]
1987	TX	"The bigger the snake, the more venom it has the potential to inject, and the more dangerous it is to people." <Authority cited: Bob Jenni, reptile aficionado>	Non-bite	BH	True		True		[187]
1975	CA	"They are dangerous at birth...more dangerous than the adult snake...very aggressive little fellows who will throw themselves into a defensive pose and strike repeatedly when disturbed. Where the more reserved adult will strike only once or twice, the baby has not yet learned control and will keep on striking, injecting more venom into his unhappy victim..." <Authority cited: None>	Non-bite	AU	False	False			[48]
1974	NM	"...the idea that a baby rattlesnake...is not as poisonous as its grandfather is erroneous. The only difference...is in the amount of poison each is capable of injecting; that grandpappy might have a more powerful squirt, but that the kid's squirt is nothing to fool around with." <Authority cited: George Biggs, Jr., biology professor at New Mexico Junior College>	Non-bite	AC	True		True		[188]
1973	CA	"...they are more dangerous than the adult snake." <Authority cited: None>	Non-bite	AU	False				[189]
1971	CA	"...baby rattlers which do not have rattles to identify them but who are able to inflict bites as lethal as those of an adult snake." <Authority cited: None>	Bite	AU	False				[190]
1969	SD	"Are baby rattlesnakes more deadly than the adult? Drop for drop, the venom of the baby is identical with that of an adult, but you get so much less of it due to the small size of the glands. The fangs are yet tiny and cannot penetrate very deep and the weight and muscular development of the baby cannot push the fangs through leather or even heavy cloth." <Authority cited: None>	Non-bite	AU	True	False		X	[191]
1968	AL	"A young rattler is considered to be more dangerous than an old one. The reason is that the old snake will try to save some of the venom for future use. The ambitious youngster will let you have the full load. <Authority cited: None> [MORE: boy bitten by baby rattlers thought to be worms placed on fish hook]	Bite	AU	False	False			[192]
1968	TX	"A small rattlesnake is much more deadly than a large rattlesnake…The	Non-bite	BH	False			X	[193]

		smaller rattlesnake's venom is much more toxic." <Authority cited: Bill Randsberger, snake handler from Sweetwater>							
1967	CA	"…baby rattlers…are even more dangerous than adult rattlers because when they come out of hibernation their venom is highly concentrated." <Authority cited: Edith Kinucan, part-time naturalist in Arcadia's Wilderness Park>	Non-bite	AS	False			X	[49]
1967	TN	"Some experts have pointed out…that while a small, young rattlesnake will inject all his venom into whatever it bites, the older, larger ones will inject only about ¼ on the first strike, holding plenty in reserve in case other strikes are indicated. This means…that a bite from a smaller snake might well be more dangerous than one from a larger reptile." <Authority cited: Gene Allbritton, salesman for New York Life Insurance Co.> [Reprint of story in Giles Free Press, May 24, 1967, in Pulaski, Tennessee]	Non-bite	OT	False	False			[39]
1966	CA	"In general, it can be said that the larger the snake the greater the danger. A larger snake will inject more venom and can stab deeper with its fangs." <Authority cited: None>	Non-bite	AU	True		True		[194]
1966	CA	"…baby rattlesnakes are nearly as dangerous as the adult…" <Authority cited: William G. Waldron, consulting public health entomologist for the County Health Department>	Non-bite	HP	True				[195]
1965	CA	"Young rattlers are more dangerous than mature snakes…because they are poisonous from the time they are born and will strike at almost anything." <Authority cited: Police Chief Ray McLean>	Non-bite	FP	False				[50]
1965	ID	"The bigger the snake the more dangerous the bite because of longer fangs and more venom." <Authority cited: Pocatello Doctors>	Non-bite	HP	True		True		[196]
1965	TX	"The larger the snake, the more dangerous it is, because of the increased volume of venom it carries." <Authority cited: None>	Non-bite	AU	True		True		[197]
1962	CA	"The infant has less poison in its poison sacs, but what is there is just as venomous." <Authority cited: William G. Waldron, Los Angeles County Health Department>	Non-bite	AU	True		True		[198]
1962	Alberta, Canada	"The larger the rattlesnake the more venom it probably carries so the more dangerous its bite would be." <Authority cited: None>	Non-bite	AU	True		True		[199]
1961	CA	"Generally speaking, the larger the snake the greater is the amount of poison injected." <Authority cited: None>	Non-bite	AU	True		True		[200]
1958	CA	"…baby rattlesnakes are more dangerous than older ones because they have no rattles and make no noise." <Authority cited: None>	Non-bite	AU	False				[201]
1938	NE	"...the larger the snake the larger the dose of venom by the bite." <Authority cited: None>	Non-bite	AU	True		True		[202]
1937	TX	"The larger the snake, the more dangerous the bite is the rule because larger snakes have larger venom sacs and inject more venom."	Non-bite	AU	True		True		[203]

		<Authority cited: None>							
1936	FL	“Contrary to popular belief…the bite of a baby rattler is more dangerous than that of an adult snake. The venom is thinner and far more important.” <Authority cited: Roy L. Montgomery, owner of the Reptile Leather company>	Bite	BH	False			X	[51]
1930	KY	“…the one which can give you the biggest dose is the one to be most feared. Therefore, a big copperhead would be more dangerous than a little rattlesnake—a big rattlesnake would be worse than a little moccasin—and so on. <Authority cited: None>	Non-bite	AU	True		True		[204]
1929	PA	“Of course, the longer the fangs, the more dangerous is the bite, because the venom is with greater certainty thrown into the arterial bloodstream.” <Authority cited: None>	Non-bite	AU	True				[52]
1927	TX	“The bigger the snake, the better the milker he is. They have large poison sacs and consequently yield more venom.” <Authorities cited: Leonard Keeler and E. L. Woolsey, medical students>	Non-bite	HP	True		True		[205]
1925	IN	“The bite from a large snake is more dangerous than that from a small one.” <Authority cited: C. P. Fordyce>	Non-bite	OT	True				[206]
1923	WI	“…the larger they were, and therefore the more dangerous…” <Authority cited: None>	Non-bite	AU	True				[207]
1909	KS	“The smaller the snake, the less deadly it is. Its fangs are not so long, and its glands do not secrete so much venom.” <Authority cited: book by Raymond Lee Ditmars, curator of reptiles in the New York Zoological park>	Non-bite	ZM	True		True		[208]
1907	NE	“The diamondbacks of the south attain a much larger size, and consequently inject more venom and their bite is proportionately more dangerous. <Authority cited: Francis Metcalf, in Outing, “Some Rattlesnake Fallacies”>	Non-bite	OT	True		True		[209]
1907	TX	“A bite from any venomous snake is dangerous, in proportion to the size of the snake and the amount of venom that enters the circulation.” <Authority cited: None>	Non-bite	AU	True		True		[210]
