# Supplementary material for: Are Baby Rattlesnakes More Dangerous than Adults? Origin, Transmission, and Prevalence of a Media-Driven Myth, with Evidence of Effective Messaging to Dispel It
Source: Toxins (Basel). 2026 Mar 14;18(3):144. doi: 10.3390/toxins18030144 (PMC13030322; doi:10.3390/toxins18030144)
Supplement: Supplementary file 1 [file toxins-18-00144-s001.zip › SUPPLEMENTAL TABLE S2.pdf]

Supplemental Table S2. Responses of United States university students in introductory biology courses, by region, to a survey question about familiarity with the babies-more-dangerous myth and the venom-dump hypothesis. The instructor asked: "*Have you heard that baby rattlesnakes are more dangerous than adults because they have not learned to control the amount of venom they inject when biting, and therefore inject more?*" The surveys were conducted from September 2008 to March 2009. One class was a high school.

INSTITUTIONS	STATE	Familiar	Sampled	Percent
Washington State University TriCities	WA	10	38	26.3
Walla Walla University	WA	66	94	70.2
Wenatchee Valley College	WA	20	48	41.7
The University of Montana - Western	MT	20	27	74.1
University of Idaho	ID	50	144	34.7
College of Southern Idaho	ID	15	34	44.1
The College of Idaho	ID	39	84	46.4
**NORTHWEST (N = 7)**	**3 states**		**AVG:**	**48.2**
Pacific Union College	CA(N)	53	74	71.6
Sacramento City College	CA(N)	38	83	45.8
Antelope Valley	CA(S)	67	114	58.8
Crafton Hills	CA(S)	49	68	72.1
La Sierra University	CA(S)	61	162	37.7
Mt. San Antonio	CA(S)	21	43	48.8
Truckee Meadows Community College	NV	15	40	37.5
Northern Arizona University	AZ	43	78	55.1
New Mexico Institute of Mining and Technology	NM	13	26	50
New Mexico Tech	NM	14	29	48.3
**SOUTHWEST (N = 10)**	**4 states**		**AVG:**	**52.6**
Northern State University	SD	13	38	34.2
St. Mary's University	MN	2	25	8
Upper Iowa University	IA	6	24	25
Clarke College	IA	1	13	7.7
Union College	NE	22	38	57.9
Nebraska Wesleyan University	NE	2	20	10
Neosho County Community College	KS	10	22	45.5
Bethel College	KS	4	39	10.3
Truman State University	MO	3	24	12.5
**NORTH-CENTRAL (N = 9)**	**6 states**		**AVG:**	**23.4**
Northwestern Oklahoma State University	OK	41	79	51.9
Cameron University	OK	42	107	39.3
Arkansas State University	ARK	39	166	23.5

Clarendon College	TX	42	68	61.8
McMurray University	TX	31	57	54.4
St. Mary's University	TX	9	54	16.7
Amarillo College	TX	55	132	41.7
Sam Houston State	TX	20	63	31.7
Southwestern Adventist University	TX	18	55	32.7
Nicholls State University	LA	19	75	25.3
**SOUTH-CENTRAL (N = 10)**	**4 states**		**AVG:**	**37.9**
Andrews University	MI	40	112	35.7
Kettering College of Medical Arts	OH	2	33	6.1
Atlantic Union College	MA	10	39	25.6
State University of New York at Albany	NY	8	63	12.7
Mercer County College	NJ	19	136	14
The College of New Jersey	NJ	2	35	5.7
Columbia Union College	MD	0	14	0
Marshall University	WV	47	151	31.1
**NORTHEAST (N = 8)**	**7 states**		**AVG:**	**16.4**
Southern Adventist University	TN	42	104	40.4
Clemson University	SC	37	103	35.9
Oakwood University	AL	13	41	31.7
Huntingdon College	AL	10	43	23.3
Troy University	AL	85	234	36.3
University of West Georgia	GA	50	156	32.1
University of West Florida	FL	15	50	30
Florida Hospital College of Health Sciences	FL	4	26	15.4
Lecanto High School	FL	37	126	29.4
**SOUTHEAST (N = 9)**	**5 states**		**AVG:**	**30.5**

**TOTAL COLLEGES/HIGH SCHOOLS**	**STATES**	**TOTAL STUDENTS**		**AVG**
**53**	**29**	**1394**	**3751**	**37.2**
